# Systemic inflammatory perturbations triggered by neuropathic pain in L5 compressed mouse and rat model

**DOI:** 10.1016/j.jot.2025.10.006

**Published:** 2025-12-23

**Authors:** Shangmin Chen, Zhikai Zheng, Hua Ying, Fang Ye, Peng Liao, Jian Zhou, Sihan Tong, Junjie Gao, Delin Liu, Zhigang Zhong, Yi-Gang Huang

**Affiliations:** a Sports Medicine Center, The First Affiliated Hospital of Shantou University Medical College, Shantou, 515041, China; b Sports Medicine Institute, Shantou University Medical College, Shantou, 515041, China; c Department of Orthopaedics, Shanghai Sixth People's Hospital Affiliated to Shanghai Jiao Tong University School of Medicine, Shanghai, 200233, China; d Institute of Microsurgery on Extremities, Shanghai Sixth People's Hospital Affiliated to Shanghai Jiao Tong University School of Medicine, Shanghai, 200233, China; e Shanghai Key Laboratory of Orthopaedic Implants, Department of Orthopaedic Surgery, Shanghai Ninth People's Hospital, Shanghai Jiao Tong University School of Medicine, Shanghai, 200011, China; f Bone Marrow Transplantation Center of the First Affiliated Hospital & Liangzhu Laboratory, Zhejiang University School of Medicine, Hangzhou, Zhejiang, 310000, China; g Department of Medicine, The University of Hong Kong, Hong Kong Special Administrative Region of China

**Keywords:** lymphocyte, Macrophage, Nerve compression, Neuroinflammation, Neuropathic pain, Single-cell RNA-sequencing

## Abstract

**Background:**

Neuropathic pain is caused by lesions or disease affecting the somatosensory nervous system either in the periphery or centrally. Unresolvable inflammation is one of the main causes of the difficulty in managing prolonged pain. Although neuropathic pain is characterized by local inflammatory infiltration at the lesion site, whether neuropathic pain can induce systemic inflammation and the underlying mechanisms remain unknown.

**Methods:**

The systematically effects of neuropathic pain was we investigated by using lumbar 5 (L5) nerve compression mouse and rat model and performed multi-omic analysis on multiple organ systems at three levels: (1) local compressed nerves (L4-6), (2) brain and bone marrow, and (3) major indirect organs (including heart, liver, lung, kidney, colon, small intestine, spine and spleen).

**Results:**

Bulk RNA sequencing of nerves (L4-6) revealed L5 compression resulted in inflammatory response, metabolic disorders, neuron regeneration. Single-cell RNA sequencing of bone marrow and brain cells identified perturbations in neutrophils and macrophages within the bone marrow, and in microglia within the brain, highlighting the upregulation of inflammatory and immune response pathways. Further ATAC sequencing of bone marrow-derived macrophages revealed upregulation of Rho protein signal transduction and small GTPase-mediated signal transduction. Bulk RNA sequencing of major organs (heart, liver, lung, kidney, colon, small intestine, spine and spleen) revealed activated immune and dysregulated lipid metabolism, with macrophages playing a key role in the process through the activation of different pathways.

**Conclusion:**

Our study reveals that nerve compression-induced neuropathic pain triggers systemic inflammation, characterized by upregulated expression of pro-inflammatory genes (IL13 and Csf3 in bone marrow and brain; TNF-α in the compressed nerve), altered chromatin accessibility in bone marrow macrophages, increased aerobic respiration and energy metabolism in the lungs, hepatic metabolic dysfunction, and renal lipid accumulation. Additionally, ligand-receptor networks facilitate cross-organ inflammation. This atlas redefines neuropathic pain as a multi-organ disorder and identifies myeloid-immune signaling pathways as potential therapeutic targets.

**The translational potential of this article:**

Our study identifies key genes and signaling pathways that may contribute to systemic inflammation in nerve compression-induced neuropathic pain. We sorted out potential intervention targets to modulate the inflammatory process in neuropathic pain, such as inhibitors of Csf3 and IL13, as well as targeting the IL6/TNF-α pathway. However, further functional validation is necessary to confirm their therapeutic efficacy.

## Graphical abstract

**Figure:**
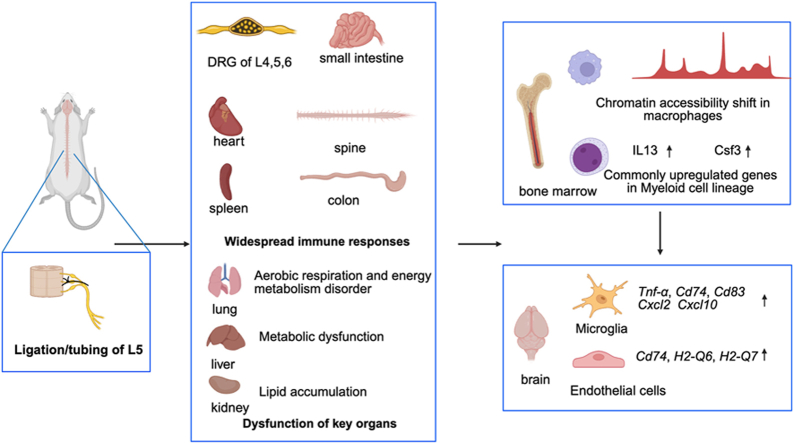

## 1 Introduction

Neuropathic pain refers to pain that originates from pathology of the nervous system, and is affecting 7–9 % of the general population [1]. Classical neuropathic pain includes trigeminal neuroglia and traumatic neuropathy, Painful diabetic neuropathy, and postherpetic neuralgia. Patients often describe neuropathic pain as burning, lightning-like with hyperalgesia. Management of neuropathic pain is challenging. It cannot simply relieved by nonsteroidal drugs, different from the nociceptive pain (pain that originate from nonneural tissues) [2]. This is because of the complicated mechanism of neuropathic pain.

Immune response plays a key role in production of neuropathic pain. The recruited immune cells induce alterations in ion channels, metabolites, glial-derived mediators [2]. For example, macrophages were recruited and activated in the lesion site [3]. The activated macrophages decreased current threshold of neuron by releasing inflammatory mediators (such as prostaglandin E2, serotonin, and adenosine). These activated immune mediators lead to a central hypersensitization, and producing increased current amplitude and a hyperpolarizing shift of its activation curve, to develop the radicular pain [[3], [4], [5], [6]]. Immune activation is a key feature of neuropathic pain. However, it is still unclear how immune cells are initiated in the bone marrow and the systemic effects following immune activation in neuropathic pain.

Here, we hypothesize that local nerve damage may trigger systemic inflammation, involving bone marrow immune activation, neuroinflammation, and immune responses in major organs. We aim to depict immune cells initiated in bone marrow and the systematic results after immune activation following neuropathic pain. Our group previously established an L5 nerve compression model, and rats showed classical pain behavior 7 days after surgery [7]. This study uses the same method to establish nerve compression in both mouse and rat models. We performed sequencing analysis on the major organs, and found the lumbar nerve root compression not only induces inflammation at lesion location, but also triggers a whole-body inflammation status. The bioinformatic analysis reveals that macrophages and T lymphocytes at the compressed site, as well as microglial cells in the brain, play key roles in the initiation and progression of neuroinflammation. We also identified a list of the regulatory molecules of inflammation which play a role in onset of neuropathic pain after compression. Taken together, our study provides a gene atlas of inflammation after nerve root compression and insights into cellular heterogeneity of neuropathic pain.

## 2 Methods

### 2.1 Animals

All procedures involving mice and rats were approved by ethics committee of the Shanghai Jiao Tong University Affiliated Sixth People's Hospital (No. DWLL2024-0657). The L5 nerve compression mouse model was established following protocols described in previous studies [8,9]. In brief, 8-week-old male C57BL/6J mice and 8-week-old male Sprague Dawley rats were administered isoflurane anesthesia, followed by a 2 cm incision 1 mm to the left of the midline, running parallel to the vertebral spines. The muscles covering the transverse process of the L6 vertebra were then excised, and a portion of the L6 transverse process was removed to expose the L4 and L5 spinal nerves. Then, the left L5 compression was performed by using 6-0 silk suture ligation or embedding polyethylene capillary tube (0.35 mm inner diameter and 0.45 mm outer diameter) without damaging the L4 spinal nerve. In the mouse experiments, 8-week-old male C57BL/6J mice were used. The control group (n = 3) underwent sham surgery, while the compression group (n = 3) received the nerve compression surgery. In the rat experiments, 8-week-old male Sprague Dawley rats were used. Six rats underwent sham surgery and six rats received compression surgery. Among the rats, half (n = 3) were sacrificed on Day 3 after surgery, and the remaining half (n = 3) on Day 7. All mice and rats were housed in standard cages with an SPF environment in a 12-h light/dark cycle at a room temperature of 22 °C ± 2 °C, humidity of 50 % ± 5 %, with free access to food and water.

### 2.2 Sample preparation for bulk RNA-seq sequencing

Mice were sacrificed, nerves, heart, liver, lung, kidney, colon, small intestine, spine and spleen were collected and stored in liquid nitrogen. Bone marrow cells were isolated by flushing the long bones with PBS and then passing them through a 70 μm cell strainer. Bone marrow samples were then incubated with red blood cell lysis buffer (FUSHENBIO, Cat. FS1143) for 10 min at 4 °C. Next, the lysis was stopped by adding an equal volume of PBS, and samples were centrifuged at 350 g for 5 min at 4 °C. Then, the supernatant was removed, and the samples were resuspended and incubated in DMEM supplemented with 20 % FBS on ice, in preparation for further analysis.

### 2.3 RNA-seq

Extracted RNA from nerves, heart, liver, lung, kidney, colon, small intestine, spine and spleen by using Trizol reagent (Thermofisher), quantified and purified using Bioanalyzer 2100 and RNA 6000 Nano LabChip Kit (Agilent). Following purification, mRNA library was constructed, fragmented, amplified, and loaded into the nanoarray and sequencing was performed on Illumina NovaSeq 6000 platform following the vendor's recommended protocol. After sequencing, generated reads were filtered and mapped to the reference genome using HISAT2 (v2.0.4) and assembled using StringTie (v1.3.4d) with default parameters. Then, all transcriptomes from all samples were merged to reconstruct a comprehensive transcriptome using GffCompare software (v0.9.8), and the expression levels of all transcripts were calculated by Stringtie and ballgown. Differential gene analysis was performed by DESeq2 software and then subjected to enrichment analysis of GO functions. GSEA was performed using GSEA software (version 4.1.0; Broad Institute, MIT). Genes were ranked according to their expression; gene sets were searched from website (https://www.gsea-msigdb.org).

### 2.4 Single-cell RNA-seq library preparation and sequencing

Single-cell RNA-seq libraries were prepared with Chromium Next GEM Single Cell 3′ GEM, Library & Gel Bead Kit v3 (PN-1000094) according to the manufacturer's instructions to construct the scRNA-seq library. Single cell suspension of brain and bone marrow samples was loaded onto the Chromium single-cell controller to generate emulsion containing single cell and gel beads according to the manufacturer's instructions. Cells were lysed and the released RNA was barcoded through reverse transcription in individual droplet. cDNA was amplified and sequencing libraries were constructed. Sequencing was performed on the Illumina Novaseq 6000 sequencer with 150 bp paired-end reads mode. The raw sequencing reads were processed by Cell Ranger (v.2.1.0) with the default parameter using reference genome mm10.

### 2.5 Quality control and clustering

Processed digital gene expression matrix was treated using Seurat [10] in R (version 3.6.3). Briefly, each matrix were normalized using the function “NormalizeData” with scale.factor = 10000. We filtered cells with less than 500 genes and high mitochondrial genes (>15 %). The principal component analysis was performed by the “RunPCA” function. Clustering was conducted using the “FindNeighbors” and “FindClusters” function with top 20 dimensions and resolution = 1. Marker genes for each cluster were identified with the “FindAllMarkers” function with min.pct = 0.25 and logfc.threshold = 0.25. Dimensionality reduction was performed with the “RunUMAP” function. The cell types were identified using the canonical cell-type-specific markers genes that have been reported [11,12].

### 2.6 Differentially expressed genes analysis and gene set function enrichment

For each group, we performed differentially expressed genes (DEGs) analysis of specific cell type using FindMarkers function in Seurat with logfc.threshold = 0, min.pct = 0. We filtered and calculated the number of DEGs in each cell type with threshold of absolute log fold change >1 and adjust *P* value < 0.05. For selected cell type, we performed gene function enrichment analysis using clusterProfiler package [13] with default parameters. GO enrichment analysis was also performed using Metascape [14]. All DEGs in bone marrow and brain were used to perform Gene Set Variation Analysis (GSVA) using GSVA R packages.

### 2.7 Ligand-receptor analysis between brain and bone marrow cell types

CellChat (version 1.4.0) was used to analysis of potential receptor-ligand pairings [15]. We aggregated the gene expression levels of immune cells from mouse bone marrow. Receptors and ligands expressed in more than 10 % of the cells in each cluster were considered. We used secreted signaling for cell–cell communication analysis and filtered out the communication less than 10 cells. The cutoff was set with the mean expression greater than 0.05 and P values smaller than 0.05. We used the sum of the number of receptor–ligand pairs in each cell–cell pairing to indicate the strength of the cell–cell interactions.

### 2.8 Analysis of rat bulk RNA-Seq data and ATAC-seq data

After quality control, raw sequencing reads in RNA-seq were aligned to the rat reference genome (Rnor_6.0) using STAR (version 2.7) [16] with the default settings. We used featuresCounts (version 1.6.0) [17] with the following parameters: “featureCounts -T 40 -p -t exon -g” to generate gene-level read counts. Differential gene expression levels were calculated using DEseq2 (version 1.28.1) [18] and gene annotations were obtained from Ensembl. Raw sequencing reads in ATAC-seq were mapped using bowtie2 [19]. Peak Calling was performed using MACS2 [20]. Annotation of peaks was performed using ChIPseeker [21]. Homer (http://homer.ucsd.edu/homer/download.html, v4.11.1) was used for motif analysis. We first generated the DEGs from mice scRNA-seq and rat bulk RNA-seq datasets, respectively. The DEGs between mice and rat were checked through biomaRt (version 2.54.1) before overlapping analysis.

## 3 Results

### 3.1 Bulk RNA-seq analysis of rat dorsal root ganglion at day3 and day7 after entrapment injury

We establish L5 nerve compression rat model by ligating the lumbar 5 dorsal root ganglion in rat. To identify the dynamic gene regulation in entrapment injury, we performed bulk RNA sequencing of dorsal root ganglion (L4, L5, L6) [22] in compression and control group at day3 and day7 after entrapment injury, respectively. In day3 and day7, DEGs analysis were performed between compression group and control group. Generally, L4 and L5 possessed the greatest number of DEGs (number of DEGs around 2,000, log2 fold change >1 and *p* value < 0.05). After L5 compression, we observed obvious perturbation pattern in L4 and L6 at day3. VennPlot demonstrated overlap of differentially expressed genes between L4, L5, L6 at two time point respectively. L4 and L5 shared most upregulation genes and downregulation genes (SFig. 1a–d). Sample-to-sample distance heatmap and PCA plot based on gene expression similarities also revealed obvious correlations between L4 and L5 at day3 (SFig. 1e and f).

Both L5 (compression site) and L4 at day3 shared common and quick inflammatory response based on lymphocyte proliferation (T cells) and mononuclear cell proliferation (SFig. 2d–f). KEGG pathway interaction analysis showed strong up-regulation of cytokine−cytokine receptor interaction and chemokine signaling pathway (SFig. 2e–g). In L4 and L5 myeloid cells, most of up-regulated genes were associate with pro-inflammatory state (SFig. 2a and b), while L6 demonstrated enrichment of regulatory T cells marker *Cd4* (SFig. 2c). GO terms of L6 up-regulated genes indicated MHC II antigen processing and B cell mediated immunity (SFig. 2h). Pathway enrichment of L6 suggested inflammation regulation events such as T helper cells differentiation (SFig. 2i).

**Fig. 1:**
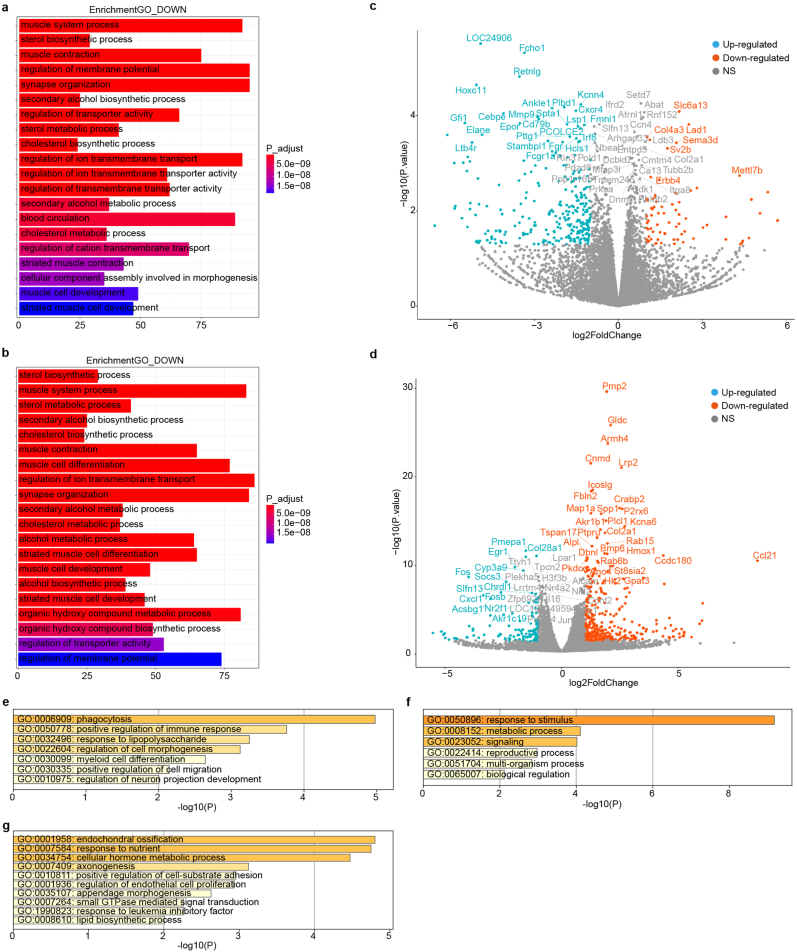
Differentially expressed genes analysis between L4 and L6 DRGs. a, **b**. GO terms of down-regulated genes in L4 at day3 (**a**) and day7 (**b**). **c**, **d**. Volcano plot showing the differentially expressed genes between L4 and L6 at day3 (**c**) and day7 (**d**) after compression. **e**, **f**. Compared with L4, the enriched GO functions of up-regulated genes in L6 at day3 (**e**) and day7 (**f**). **g**. Compared with L6, the enriched GO functions of up-regulated genes in L4 at day7.

Function enrichment terms of down-regulated genes in L4 at two time point both involved multiple metabolic process, myelination and synapse organization (Fig. 1a and b). Thus, a direct inflammatory and neuron injury response is persistent in these two locations. In contrast, we identified fewer DEGs (around 200) in L6. Next, we compared the DEGs between L4 and L6. At day3, L6 highly expressed myeloid cell marker Elane, Irf8 and B cell marker Cd79b (Fig. 1c). Compared with L4, Gene ontology enrichment of up-regulated gene in L6 showed myeloid cell differentiation and positive regulation of immune response (Fig. 1e). At day7, L4 uniquely expressed myelin gene Pmp2, Fbln2, Hmox1 that functional related to metabolic process and stimulation response [23,24] (Fig. 1d–f). Upregulation gene functions in L6 were enriched in cellular hormone metabolic process (Fig. 1g).

### 3.2 Molecular heterogeneity of bone marrow and brain after L5 nerve compression

**Fig. 2:**
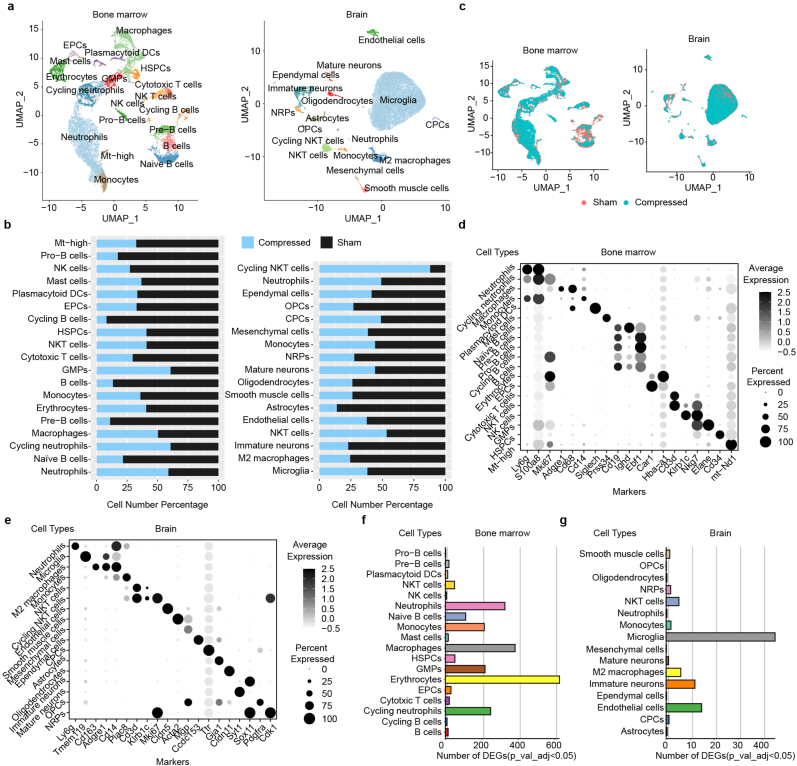
Single cell transcriptome landscape of mouse bone marrow and brain under entrapment injury. a. UMAP plot of bone marrow and brain cell types. **b.** Cell type ratio of bone marrow (left) and brain (right) cell types in control group and compression group (surgery). **c.** UMAP plot of bone marrow and brain cells colored by control and compression group. **d, e.** Dot plot showing the cell type specific marker genes in bone marrow (**d**) and brain (**e**) cell types. **f, g.** Number of differentially expressed genes (log2 fold change >0.5 and adjust *p* value < 0.05) in bone marrow (**f**) and brain (**g**).

To investigate the impacts of nerve compression on bone marrow and brain, we performed single cell RNA-Seq analysis on L5 nerve compression mouse. We established L5 nerve compression mouse model by embedded polyethylene capillary tubing around left L5 nerve. The mice of control group underwent a sham surgery. After seven days, both groups of mice were euthanized to collect bone marrow and brain tissues, which were then processed for 10x Genomics single-cell RNA sequencing. In total, we sequenced a total of 16,273 bone marrow cells and 19,212 brain cells. After quality control (SFig. 3a), we performed with principal component analysis and clustered the data using Uniform Manifold Approximation and Projection (UMAP) implemented in Seurat1 (Fig. 2a). As a result, brain dataset was classified into seventeen major types based on established cell markers (Fig. 2a–e, SFig. 3b), including neuron restricted precursors (NRPs, *Cdk1* and *Syt1*), immature neurons (*Sox11*), oligodendrocytes progenitor cells (OPCs, *Pdgfra*), oligodendrocytes (*Cldn11*), astrocytes (*Gja1*), choroid plexus epithelial cells (CPCs, *Ttr*), ependymal cells (*Ccdc153*), microglia (*Tmem119*), endothelial cells (*Cldn5*) and other immune cells. The cell number ratio analysis showed 7-day of L5 nerve compression decreased of glia cells and increased of NKT cells in brain (Fig. 2b and c).

For bone marrow, cells were classified into nineteen cell types based on hematopoietic lineage markers (SFig. 3c). Dot plot showed the top differentiated expressed marker gene of each cluster (Fig. 2d), including B cells (*Cd19*, *Ebf1* and *Ighd)*, cytotoxic T cells (*Cd3d*), NKT cells (*Nkg7*), NK cells (*Klrb1c*), neutrophils (*Ly6g*, *S100a8)*, macrophages and monocytes (*Adgre1, Cd14*), plasmacytoid DCs (*Siglech)*. Erythroid progenitor cells (EPCs) (*Car1*), granulocyte-monocyte progenitors (GMPs) (*Elane)*, hematopoietic stem and progenitor cells (HSPCs) (*Cd34*). The cell number ratio analysis showed B cell lineage was reduced after 7-day of L5 nerve compression (Fig. 2b and c).

### 3.3 Immune cells in bone marrow and brain strongly response to the nerve compression

**Fig. 3:**
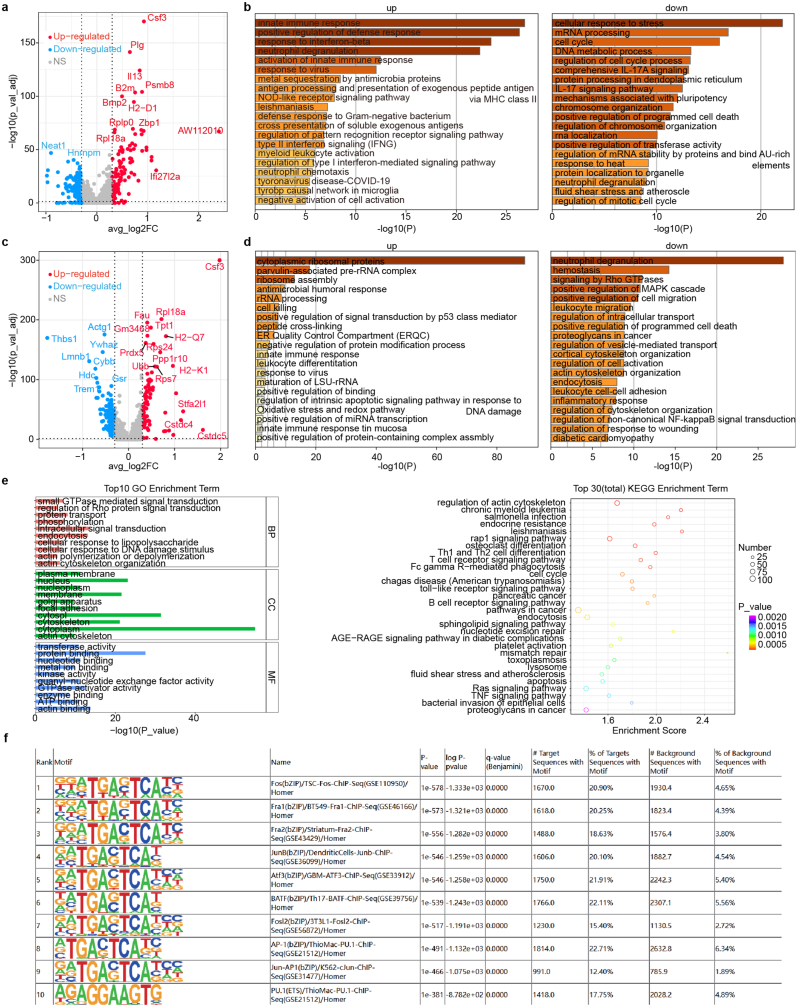
Differentially expressed genes and functions in represented bone marrow cells. a, c. Volcano plot showing the differentially expressed genes in macrophages (**a**) and neutrophils (**c**). **b, d.** Gene ontology function enrichment of up-regulated genes (left) and down-regulated genes (right) in macrophages (**b**) and neutrophils (**d**) in compression group. **e,** Gene ontology (left) and pathway (right) enrichment analysis of differentially accessible peaks in BMDM through bulk ATAC-seq. **f,** Top 10 motif motifs Enrichment results in BMDM through bulk ATAC-seq.

Next, we performed differentially expressed genes (DEGs) analysis of subclusters in bone marrow and brain. The results showed a monocyte lineage has most DEGs. In bone marrow, neutrophils and macrophages had the greatest number of DEGs (adjust *p* values < 0.05) besides erythrocytes (Fig. 2f), while in brain, microglia showed large number of regulated genes (Fig. 2g). Bone marrow myeloid cells (macrophages, neutrophils) showed common up-regulation of colony stimulating factor 3 (*Csf3*) and *IL13* after nerve compression (Fig. 3a–c). Strong expression of inflammation‐associated cytokines such as *Csf3* and *IL13* suggested a remote response in bone marrow myeloid cells [25]. The increased expression of *IL13* could also decrease the polarization of M2 macrophages in inflammation regulation [26]. This suggested the local and remote immune-mediated inflammation is associated with after nerve compression [27]. To validate this, we isolated bone marrow-derived macrophages from 8-week-old male C57BL/6J mice and treated them with 40 ng/mL TNF-α (SFig. 2g) for 24 h, followed by qPCR analysis. The results showed that TNF-α treatment upregulated the expression levels of Csf3 and IL13 in macrophages (SFig. 3d), which aligns with our transcriptional analysis findings.

Gene ontology enrichment of up-regulated genes in macrophages showed inflammatory response, innate immune response, NOD-like receptor signaling pathway and Type II interferon signaling (IFNG) after nerve injury (Fig. 3b). Down-regulation of cell cycle and IL-17 signaling pathway indicated activated regulation role of macrophage in inflammation. Neutrophils demonstrated obvious enrichment of structural constituent of ribosome, endopeptidase regulator activity and protease binding (Fig. 3d). Down-regulation of neutrophil degranulation, MAPK cascade and cell migration involved the inflammation regulation process in bone marrow. We further performed ATAC-seq of bone marrow derived macrophages (BMDM). Function enrichment of accessible peaks (most down-regulation after nerve entrapment) in BMDM highlight the regulation of Rho protein signal transduction and small GTPase mediated signal transduction during the migration, phagocytose and impaired macrophage function (Fig. 3e). The motif enrichment results suggested the inhibition of AP-1 transcription factors which represented the enhancement of inflammation regulation ability.

**Fig. 4:**
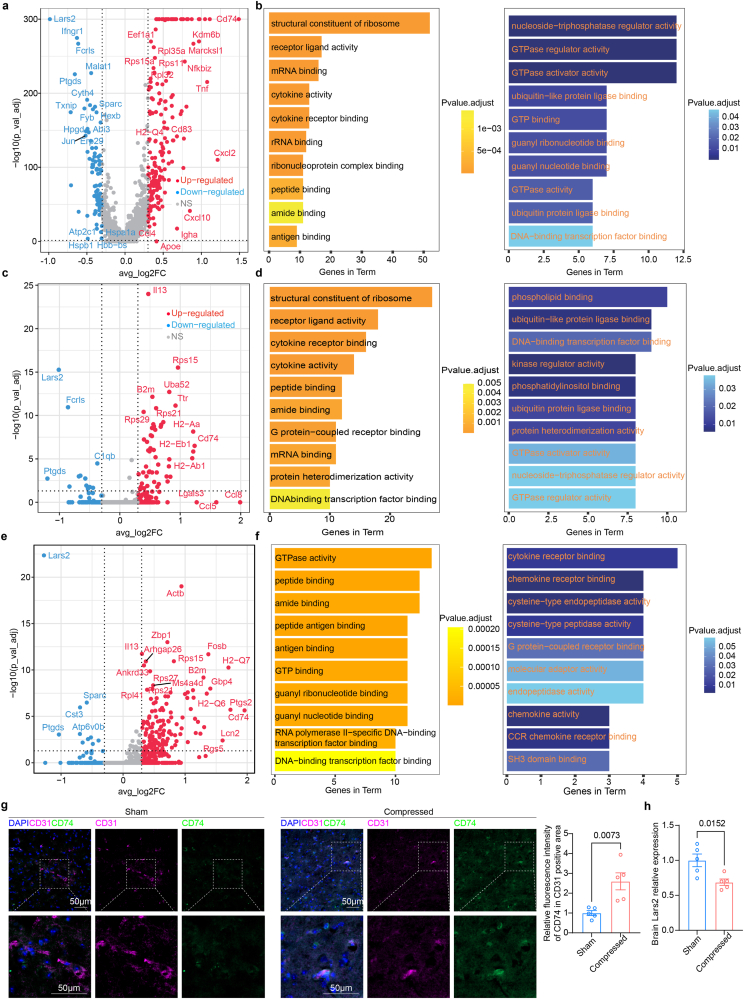
Differentially expressed genes and functions in represented brain cells. a, c, e. Volcano plot showing the differentially expressed genes in microglia (**a**), M2 macrophages (**c**) and endothelial cells (**e**). **b, d, f.** Gene ontology function enrichment of up-regulated genes (left) and down-regulated genes (right) in microglia (**b**), M2 macrophages (**d**) and endothelial cells (**f**) in compression group. **g.** Representative confocal images of brain from 9-week-old sham mice or L5 nerve compressed mice. **h.** qPCR of whole brain from9-week-old sham mice or L5 nerve compressed mice.

We further investigated the function of DEGs in brain related cells. L5 nerve root compression up-regulated expression of *Tnf-α*, *Cd74*, *Cd83* and chemokines including *Cxcl2* and *Cxcl10* in microglia (Fig. 4a). Co-stimulatory molecule *Cd83* represented the activated state of microglia [28]. Regulated M2 macrophages in brain demonstrated similar expression patterns with bone marrow macrophages (Fig. 4c). Similar gene function enrichment patterns in brain microglia and M2 macrophages indicated the formation of neuroinflammation environment based on activation of microglia (Fig. 4b–d). To describe the potential role of inflammatory signal transduction in endothelial cells. We also analyzed DEGs in brain endothelial cells (Fig. 4e). Up-regulation of MHC II gene group in endothelial cells (*Cd74*, *H2-Q6*, *H2-Q7*) indicated the immune activation state of brain endothelial cells with less cytokine receptor binding ability [29] (Fig. 4f and g). Thus, peripheral inflammatory signals could disrupt the integrity of the blood–brain barrier (BBB) through interaction between myeloid cells and endothelial cells [30]. *Lars* encoding an enzyme involved in mitochondrial protein synthesis [31] and was downregulated in multiple cell types in brain (Fig. 4a–c, e). We performed qPCR analysis of whole brain tissue after 7 days L5 nerve compression mice and control mice. The results showed that Lars2 was downregulated in brain tissue following neuropathic pain, suggesting neuropathic pain is associated with energy production disruption in brain.

**Fig. 5:**
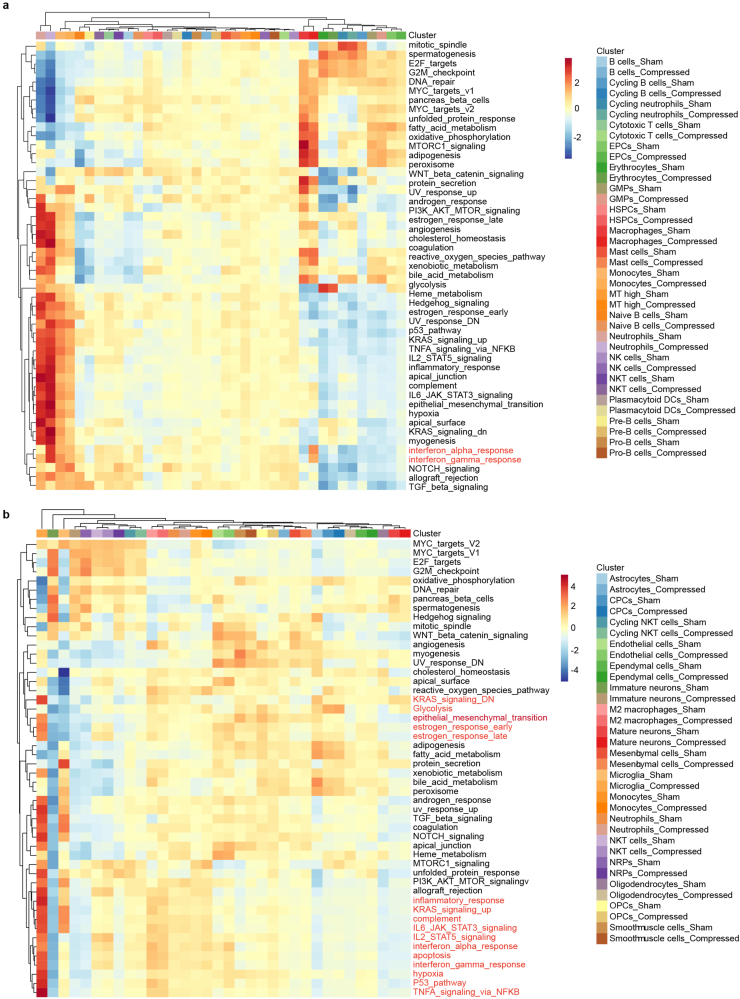
Gene set variation analysis (GSVA) of differentially expressed genes in bone marrow and brain. a, b. Heatmap showing the GSVA pathway enrichment results in bone marrow (**a**) and brain (**b**) cell types.

We wanted to further clarify the function of perturbed genes in compression group. Gene set variation analysis (GSVA) of enriched pathways were compared between compression and control condition. We observed obvious enrichment pattern of interferons alpha and gamma response in neutrophils and macrophages after compression treatment (Fig. 5a). Interferon-γ response prolongs and heightens inflammatory responses in macrophages [32]. In brain microglia, up-regulation of hypoxia and glycolysis marked the unique metabolism state associated with inflammatory response [33] (Fig. 5b). A series of inflammatory response pathways including IL6-JAK-STAT3, IL2-STAT5 and TNF-α signaling pathway were enriched in microglia after compression, indicating a common cytokine network of pro-inflammatory state [34].

### 3.4 Cell communication network in nerve entrapment mouse

**Fig. 6:**
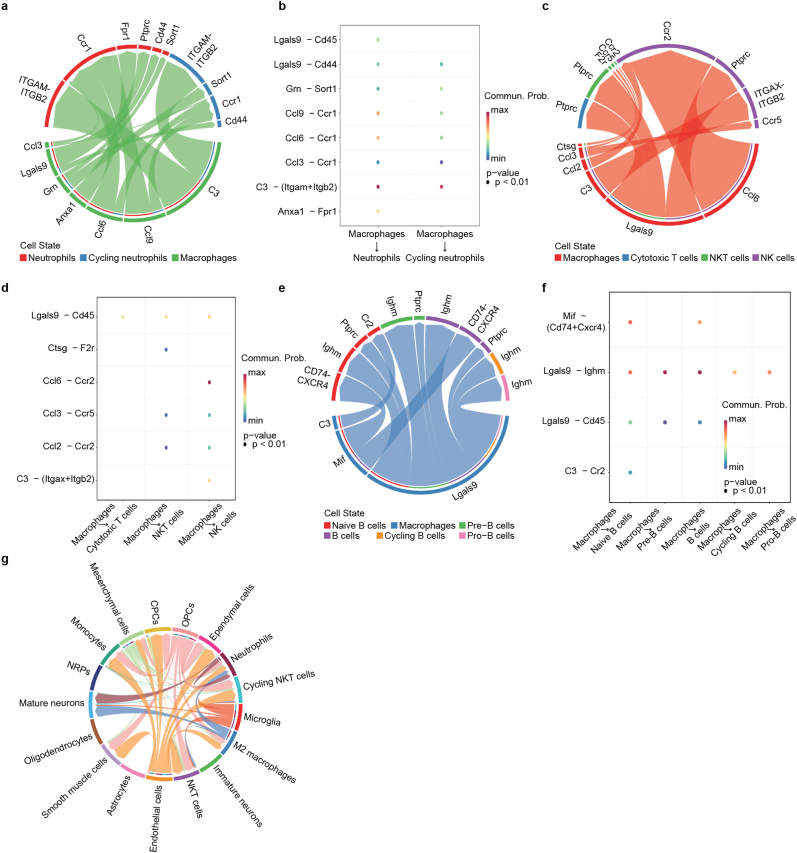
Ligand-receptor pairs analysis in bone marrow and brain. a, c, e. Network showing the ligand expressing cell type (arrow starting point) and receptor expressing cells in (**a**) Macrophages and neutrophils; (**c**) Macrophages and NK(T) cells; (**e**) Macrophages and B cells. **b, d, f.** Paired bubble plot showing the representative ligand-receptor pairs in **a**, **c**, **e**. **g.** Cxcl signaling network of ligand-receptor pairs in brain cell types.

To identify the critical cell interaction patterns and gene regulatory network during the nerve entrapment, we adopted CellChat [15] pipeline to infer the ligand-receptor pairs and gene regulatory network. Interactions between macrophages and neutrophils included complement component C3 and chemokines (*Ccl3*, *Ccl6*, *Ccl9*) (Fig. 6a and b). Ccl3-Ccr1 axis participate the recruitment and infiltration of macrophages [35]. In IL-13-induced inflammation state of bone marrow macrophages, Ccl6-Ccr1 axis may played critical role in IL-13 stimulation [36]. We also observed similar interaction patterns between macrophages, T cell and NK cells (Fig. 6c and d). Ccl3-Ccr5 axis exhibit anti-inflammatory activities by suppression of *Mmp9* in infiltrating macrophages [37]. Interaction between macrophages and B cells showed strong enrichment of Lgals9-Ighm axis (Fig. 6e and f). Galectin-9-mediated inhibition of B-cell receptor (BCR) signaling is based on the obstruction of BCR micro-cluster formation [38]. Thus, the ablation of B cell lineage and potential regulatory B cells in compression group may due to the activation of macrophages and galectin-9. We then performed global Cxcl signaling pathway network in brain cell types (Fig. 6g). Microglia, endothelial cells and OPCs secreted most chemokines as desired. Vascular endothelial cells are associated with dysfunction of BBB integrity. In summary, bone marrow derived myeloid cells together with microglia formed the neuroinflammation in compression group.

### 3.5 Comparative analysis between mouse and rat data

Compressed nerves, brain and bone marrows showed similar inflammation profile driven by myeloid lineage cells. This inspired us to explore whether specific gene families were activated, leading to the consistent inflammation pattern observed in compressed nerve, bone marrow, and brain tissues. Thus, we analyzed the overlapped DEGs between L4-6, bone marrow and brain after nerve compression (SFig. 4a). Bone marrow has highest overlapped genes with L4 and L5. GO enrichment of overlapped genes between bone marrow and L6 showed positive regulation of myeloid leukocyte mediated immunity, complement and negative regulation of lymphocytes activation which associated in inflammation regulation (SFig. 4b). In bone marrow overlapped DEGs, macrophages activation marker *Arl11* [39] and inflammatory responses inhibitor Clec12a [40] were uniquely enriched in bone marrow myeloid cells such as monocytes and neutrophils (SFig. 4c).

We then performed same pipeline on mouse brain up-regulated DEGs after compression. Patterns of overlapped genes is similar to bone marrow at day3 (SFig. 4d). Gene function of L6 co-regulated genes showed regulation of lymphocytes apoptotic process, interferon response (SFig. 4e). GO terms also indicated positive regulation of oligodendrocytes differentiation. Similar number of overlapped genes was identified in day7 datasets (SFig. 4f). Besides inflammatory response, long-term compression injury also associates with cell apoptotic and inhibited cell proliferation (SFig. 4g). We then checked the expression of L6-overlapped regulatory genes in brain cell types. Up-regulation of *Slpi* in compression group could inhibit the activation of pro-inflammatory monocytes [41] (SFig. 4h). Up-regulation of *Dusp5* is involved in the miR-32-5p-mediated effects to compete the neuropathic pain and neuroinflammation [42]. Another up-regulated genes *Tnfrsf12a* in glia*, NRPs* and other cell types is involved in pro-fibrogenic pathways after injury [43].

### 3.6 Expression patterns of pain-related genes

Neuropathic pain is correlated with neuroinflammation [44]. To identify the expression of Pain threshold related genes in nerve injury inflammation environment. We analyzed the expression of pain related genes in mouse single cell data. Mechanosensitive cation channel proteins *Piezo1* is highly expressed in DRGs to mediate mechanical pain and pain allergy [45]. In bone marrow, *Piezo1* is up-regulated in cycling cells from compression group, which may involve in injury response and repair (SFig. 5a). In brain, *Piezo1* is up-regulated in endothelial cells and CPCs. *Piezo1* expression in vascular system cells induced inflammatory signaling [46]. Up-regulation of IFN-gamma in brain NKT cells represented strong innate immune response in compression group [47]. Neuropathic pain associated gene *Ehmt2* is down-regulated in OPCs and immature neurons, which may contribute to the regulation of pain threshold [48].

Chemokine receptors *Ccr2* and *Cx3cr1* are up-regulated in monocytes and oligodendrocytes respectively (SFig. 5b). Up-regulation of ciliary neurotrophic factor receptor *Cntfr* in mature neurons indicated enhanced neuroinflammatory response [49], while down-regulation of *Cntfr* in OPCs may inhibit the survival and differentiation of oligodendrocytes [50]. Enhanced expression of potassium channel *Kcnk12* in neurons is associated with early-stage inflammation-induced pain [51]. Another neuropathic pain inducer *Gadd45a* is down-regulated in oligodendrocytes and OPCs, which could reduce the apoptosis of neurons and increases pain tolerance [52].

### 3.7 Pan-tissue transcriptome perturbations after nerve compression

**Fig. 7:**
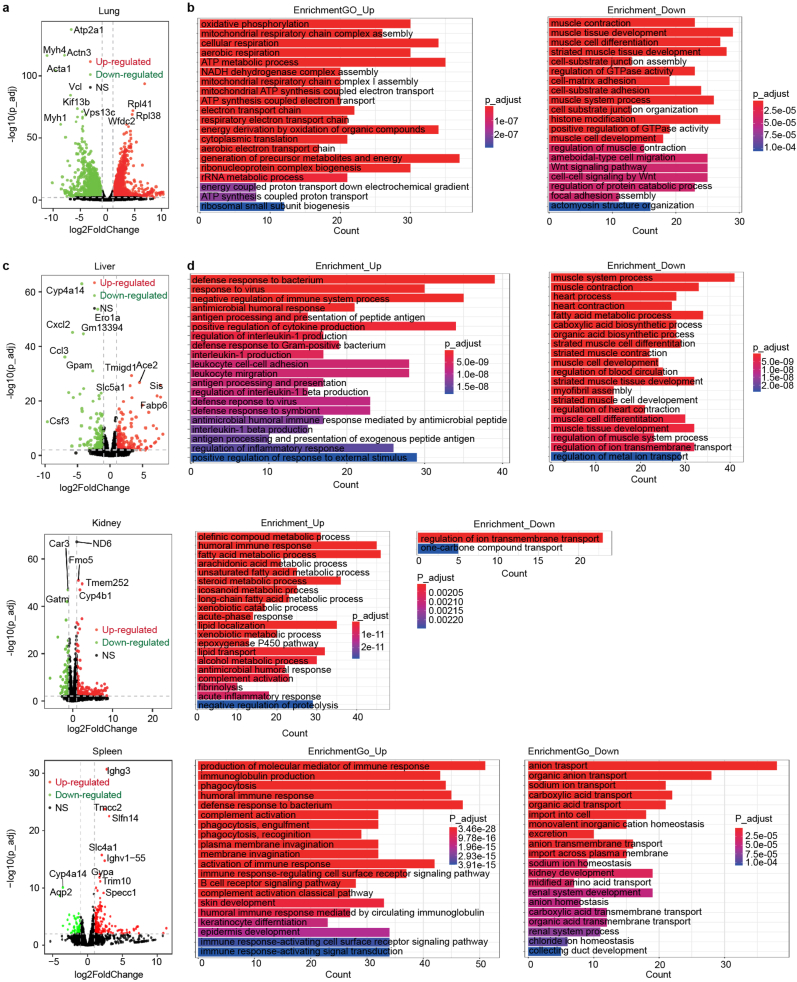
Cross-tissue transcriptome changes. a, c, e, g. Volcano plots showing the DEGs in different organs. **b, d, f, h.** Gene function enrichment of up-regulated gene (left) and down-regulated genes (right) in nerve entrapment group.

Besides the primary organs of the nervous and immune systems, we are keen to investigate whether systemic major organs exhibit a transcriptomic response during the process of nerve entrapment. Consequently, we performed RNA-seq analysis of the 8 major organs throughout the mouse body (heart, liver, lung, kidney, colon, small intestine, spine and spleen). Generally, the cross-tissue transcriptional analysis revealed major perturbations in heart, liver, lung, kidney and spleen, while lung exhibited the greatest number of variable genes after normalization (SFig. 6a). The spinal cord and small intestine exhibited modest changes at the transcriptomic level, whereas the colon and heart presented with some differentially expressed genes, albeit without significant functional enrichment (SFig. 6b). In the lung, the most prominent upregulated changes center around a more vigorous aerobic respiration and energy metabolism, encompassing oxidative phosphorylation, ATP production, and rRNA metabolism. The GTPase activity, muscle function and Wnt signaling pathway were down-regulated. Collectively, these alterations, to some extent, reflect the compensatory responses of the cardiopulmonary function under mechanical and inflammatory stimuli during nerve entrapment, as well as an increase in respiratory frequency (Fig. 7a and b).

In the metabolically relevant organ, the liver, we observed a marked downregulation of hepatic functions such as fatty acid metabolic processes and organic acid biosynthetic processes. In contrast, the production and regulation of the inflammatory cytokine IL-1β, leukocyte infiltration and migration, as well as antigen processing and presentation, were significantly enhanced in response to nerve entrapment stimulation. These findings suggest that nerve entrapment may elicit IL-1β-is associated with systemic inflammatory alterations in the liver, thereby exacerbating the pathological progression of nerve entrapment (Fig. 7c and d). In the kidney, we observed lipid dysmetabolism which was associated with lipid droplet accumulation. The lipid accumulation, together with acute phase and immune response, drive inflammation and fibrosis in chronic kidney disease [53] (Fig. 7e and f). Nerve entrapment triggered inflammation is associated with hepatocellular dysfunction and disordered lipid metabolism in the liver. Impaired lipid metabolism subsequently leads to dyslipidemia and lipid droplet accumulation within the kidney, ultimately resulting in a degree of renal injury, with the potential to progress to chronic kidney disease. In spleen, the expression of immunoglobulin genes and B cell receptor signaling pathway were elevated (Fig. 7g and h). Mapping the transcriptional alterations across diverse provincial organs reveals that the hypoxia, cellular necrosis, and subsequent inflammatory signaling cascades triggered by nerve root compression manifest not solely within the inflamed microenvironment of the central nervous system and the sustained immune activation, but also through compensatory adaptations in cardiopulmonary function and the emergence of dyslipidemia in metabolic organs including the liver and kidney. These observations imply that the pathological trajectory of nerve entrapment could precipitate systemic consequences stemming from inflammatory responses and metabolic disorder.

## 4 Discussion

The study of nerve root compression is crucial for understanding the complex mechanisms underlying neuropathic pain, a condition that affecting millions of people globally. Our study performed Bulk RNA-sequencing of the rat nerve compression model reveals neuroinflammatory changes, suggesting the involvement of immune cells such as T cell infiltration and myeloid-mediated inflammation. Then, single-cell analysis of the bone marrow–brain axis enables identification of specific cell types and demonstrates how alterations in the peripheral immune microenvironment of the bone marrow influence inflammatory infiltration in the brain via the blood–brain barrier. This links nerve compression syndrome, neuroinflammation, and neuropathic pain through the neuro–immune axis, with a particular emphasis on myeloid monocytes. In addition, Complementary ATAC-seq analysis of bone marrow-derived monocytes uncovers regulatory differences between experimental and control groups. We also describe transcriptional perturbations in other peripheral tissues and organs, indicating that these tissues may also be affected by the inflammatory immune microenvironment.

Local nerve lesion could affect remote organs. For example, our study found genes related to T cells proliferation and mononuclear cell proliferation was activated at compressed nerves. Compression in L5 nerve root remotely activate neuroinflammation by activating microglial, which expression excessive *Cxcl2* and *Cxcl10* to aggravate neuroinflammation. Additionally, potassium channel Kcnk12 in neurons is upregulated, causing an elevated K^+^ leak currents contributing to hypersensitization. In summary, our study demonstrated the nerve root compression could triggers extensive neuroinflammation that responsible for neuropathic pain.

Our work confirms the presence of local immune responses in dorsal root ganglia (DRGs), which is consistent with previous studies [8]. We further performed constructing a systemic multi-organ signaling atlas and demonstrating how immune cells are activated in the bone marrow following neuropathic pain. We also analyzed cross-tissue communication to reveal interactions between immune cells and other cell types in both the peripheral and central systems. These inflammatory networks provide potential targets for interrupting systemic inflammation. Our RNA-Seq analysis of major organs after 7 days of mouse nerve compression indicates that local nerve injury can impact distant organs, a finding consistent with human conditions. For instance, in sciatica, serum levels of IL-1β, CX3CL1, CCL2, and TNF-α are elevated [54], matching our results. Additionally, nerve compression affects lipid metabolism; sciatica has been associated with increased total cholesterol, LDL cholesterol, and triglycerides [55], which aligns with the transcriptional changes we observed in the liver and kidney.

Our study has several limitations. We primarily used a mouse model of nerve compression to investigate gene expression changes, which are inherent differences between animal models and human conditions. Therefore, translating these findings to clinical practice requires caution and further validation in human tissues and clinical studies. In our study, we utilized L5 nerve compression to mimic sciatica and peripheral nerve injury. However, neuropathic pain can be caused by other conditions such as diabetes and infectious. Further study can include multiple causes induced neuropathic pain animal models to study the biological process of neuropathic pain. We found macrophages, T cells, and Galectin-9 play key roles in neuropathic pain, but in vivo or in vitro validation is lacking. Future studies could use knockout mice or inhibitors to explore their effects. While our study identifies promising associations between immune/metabolic dysregulation and neuropathic pain, future studies using genetic knockout models or pharmacological interventions are needed to establish causal links. In addition, due to missing time-series data, the link from nerve compression to inflammatory changes in tissues remains unproven. Monitoring inflammatory indicators in clinical cohorts could help clarify this.

In conclusion, we utilized multi-tissue RNA sequencing to investigate the systemic impact of nerve entrapment pain, revealing significant gene expression changes across various organs including the heart, liver, kidneys, small intestine, spleen, and lungs. Our findings highlight the complex interplay between inflammatory and neuropathic pain mechanisms, underscoring the importance of inflammatory changes in neuropathic pain. We identified key pathways and genes involved in immune activation, metabolic dysfunction, and tissue remodeling, providing valuable insights into the systemic effects of pain, and thus offers potential therapeutic target for neuropathic pain.

## Ethics approval

All procedures involving mice were approved by ethics committee of the Shanghai Jiao Tong University Affiliated Sixth People's Hospital (Approval number: DWLL2024-0657).

## Availability of data and materials

Datasets underlying the study are available under reasonable requests to the corresponding author.

## Author contributions

Y.G.H, Z.G.Z., D.L.L. provided the essential ideas and designed the experiments. S.M.C., H.Y., F.Y., P.L. performed the research, D.L.L., J.Z., S.H.T., P.L. provided suggestions on experiments. S.M.C., H.Y., F.Y. analyzed the data and drafted the manuscript. F.Y. and J.J.G. revised the manuscript.

## Funding

This study was performed with the support of the Shanghai Frontiers Science Center of Degeneration and Regeneration in Skeletal System (BJ1-9000-22-4002).

## Declaration of Generative AI in Scientific Writing statement

The AI declaration does not apply to the use of basic tools, such as tools used to check grammar, spelling, and references. If you have nothing to disclose, you do not need to add a statement.

## Declaration of competing interest

The authors declare that they have no competing interests.
