# Supplementary material for: Systemic inflammatory perturbations triggered by neuropathic pain in L5 compressed mouse and rat model
Source: J Orthop Translat. 2025 Dec 23;56:101014. doi: 10.1016/j.jot.2025.10.006 (PMC12988534; doi:10.1016/j.jot.2025.10.006)
Supplement: Multimedia component 1 [file mmc1.pdf]

SFig.1

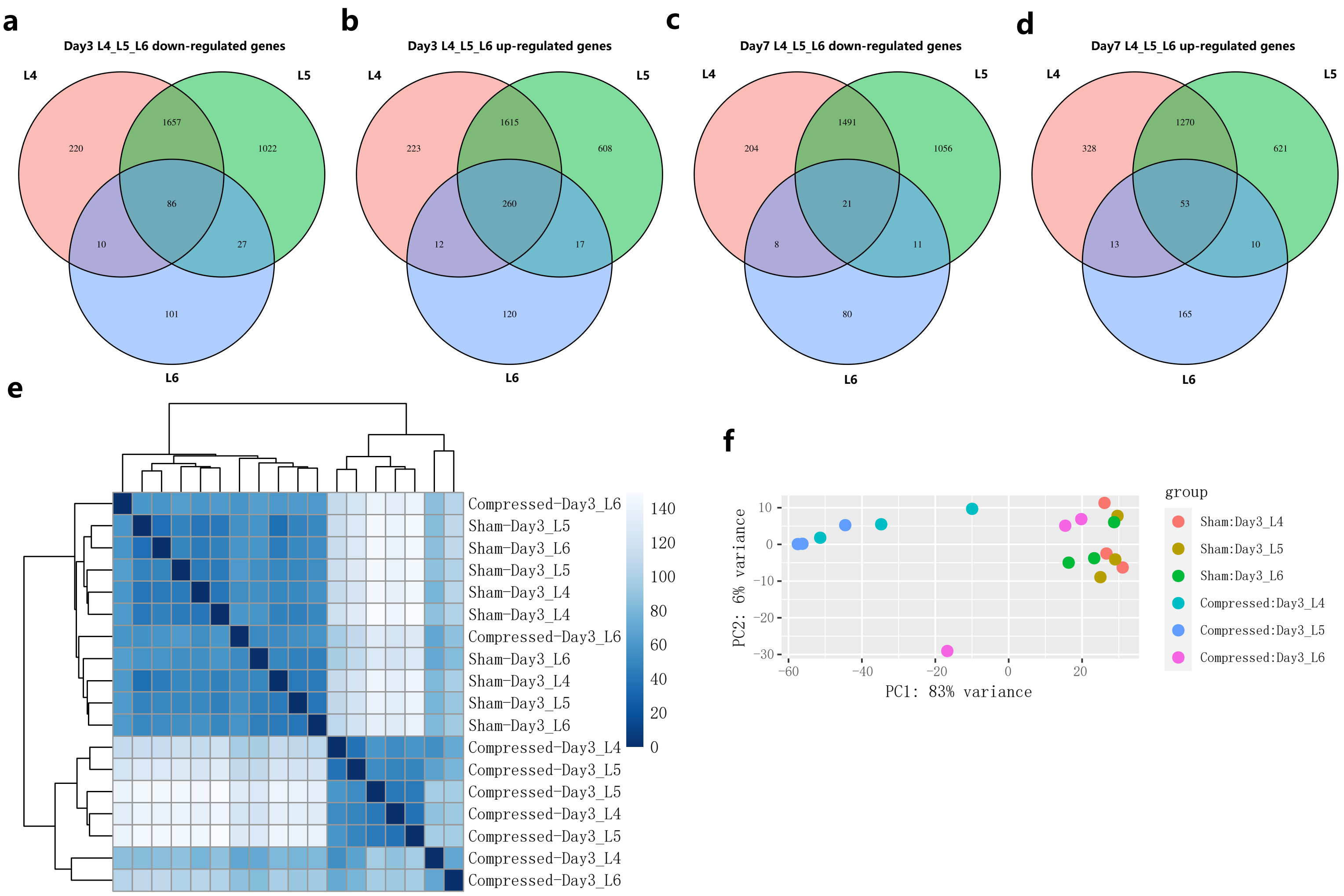

SFig.1 Overlapped genes between L4, L5 L6. a, **c**. Venn plot showing the down-regulated genes between L4, L5 and L6 at day3 (**a**) and day7 (**c**). **b**, **d**. Venn plot showing the overlapped up-regulated genes between L4, L5 and L6 at day3 (**b**) and day7 (**d**). **e**. Heatmap of the sample-to-sample similarities between L4, L5 and L6 at day3. **f**. Principal component plot of L4, L5 and L6 bulk RNA-seq samples at day3.

SFig.2

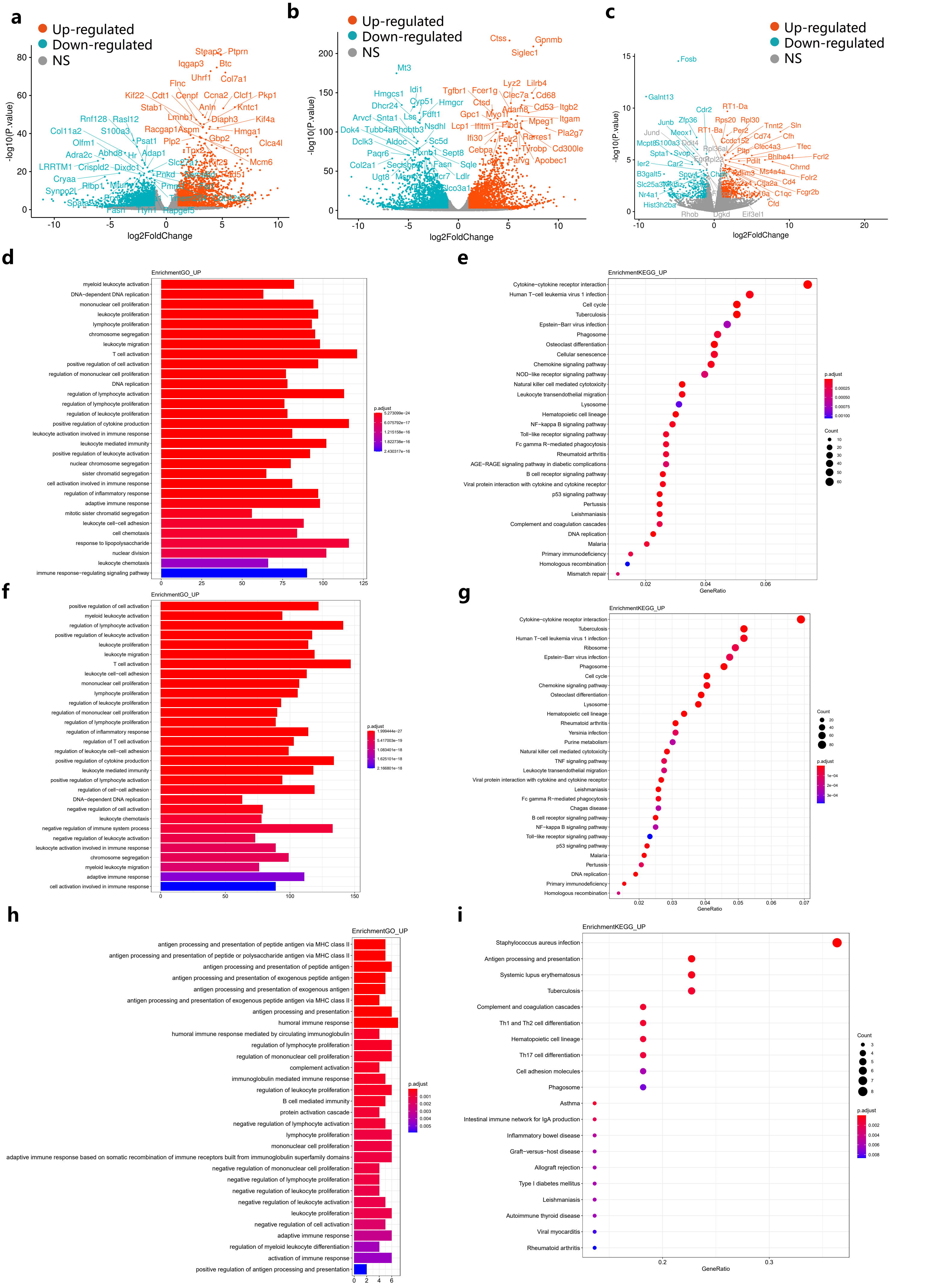
a
Up-regulated
Down-regulated
NS
-log10(P.value)
log2FoldChange
Steap2
Ptprn
Iqgap3
Btc
Uhrf1
Col7a1
Flnc
Kif22
Cdt1
Cenpf
Ccna2
Clcf1
Pkp1
Stab1
Anln
Kntc1
Rnf128
Rasl12
Lmnb1
Diaph3
Kif4a
Col11a2
S100a3
Racgap1
Aspm
Hmga1
Olfm1
Psat1
Plp2
Gbp2
Clca4l
Adra2c
Abhd8
Hr
Adap1
Gpc1
LRRTM1
Crispld2
Dixdc1
Mcm6
Cryaa
Pnkd
Synpo2l
Fasn
Ttyh1
Rapgef5
b
Up-regulated
Down-regulated
NS
-log10(P.value)
log2FoldChange
Ctss
Gpnmb
Siglec1
Mt3
Idi1
Hmgcs1
Tgfbr1
Fcer1g
Lyz2
Lilrb4
Cyp51
Hmgcr
Clec7a
Cd68
Dhcr24
Ctsd
Adam8
Cd53
Itgb2
Fdft1
Arvcf
Snta1
Lss
Gpc1
Myo1f
Mpeg1
Itgam
Dok4
Tubb4a
Rhobtb3
Nsdhl
Lcp1
Ifitm1
Plbd1
Pla2g7
Ifi30
Fcrl2
Rarres1
Dclk3
Aldoc
Sc5d
Cebpa
Tyrobp
Cd300le
Paqr6
Plxnb1
Sept8
Col2a1
Fasn
Sqle
Parvg
Apobec1
Ugt8
Ldlr
Slco3a1
c
Up-regulated
Down-regulated
NS
-log10(P.value)
log2FoldChange
Fosb
Galnt13
Cdr2
RT1-Da
Jund
Zfp36
Rps20
Rpl30
Meox1
RT1-Ba
Per2
Tnnt2
Sln
Cd74
Cfh
Mcpt8
S100a3
Ddit4
Ccdc152
Clec4a3
Tfec
Rpl36al
Plp
Fcrl2
Spta1
Svop
Bhlhe41
Ier2
Car2
Pdilt
Chrnd
B3galt5
Ms4a4a
Folr2
Slc25a3
Cd4
Nr4a1
C1qc
Fcgr2b
Hist3h2ba
Cfd
Rhob
Dgkd
Eif3el1
d
EnrichmentGO_UP
myeloid leukocyte activation
DNA−dependent DNA replication
mononuclear cell proliferation
leukocyte proliferation
lymphocyte proliferation
chromosome segregation
leukocyte migration
T cell activation
positive regulation of cell activation
regulation of mononuclear cell proliferation
DNA replication
regulation of lymphocyte activation
regulation of lymphocyte proliferation
regulation of leukocyte proliferation
positive regulation of cytokine production
leukocyte activation involved in immune response
leukocyte mediated immunity
positive regulation of leukocyte activation
nuclear chromosome segregation
sister chromatid segregation
cell activation involved in immune response
regulation of inflammatory response
adaptive immune response
mitotic sister chromatid segregation
leukocyte cell−cell adhesion
cell chemotaxis
response to lipopolysaccharide
nuclear division
leukocyte chemotaxis
immune response−regulating signaling pathway
p.adjust
5.273099e−24
6.075792e−17
1.215158e−16
1.822738e−16
2.430317e−16
e
EnrichmentKEGG_UP
Cytokine−cytokine receptor interaction
Human T−cell leukemia virus 1 infection
Cell cycle
Tuberculosis
Epstein−Barr virus infection
Phagosome
Osteoclast differentiation
Cellular senescence
Chemokine signaling pathway
NOD−like receptor signaling pathway
Natural killer cell mediated cytotoxicity
Leukocyte transendothelial migration
Lysosome
Hematopoietic cell lineage
NF−kappa B signaling pathway
Toll−like receptor signaling pathway
Fc gamma R−mediated phagocytosis
Rheumatoid arthritis
AGE−RAGE signaling pathway in diabetic complications
B cell receptor signaling pathway
Viral protein interaction with cytokine and cytokine receptor
p53 signaling pathway
Pertussis
Leishmaniasis
Complement and coagulation cascades
DNA replication
Malaria
Primary immunodeficiency
Homologous recombination
Mismatch repair
GeneRatio
p.adjust
Count
f
EnrichmentGO_UP
positive regulation of cell activation
myeloid leukocyte activation
regulation of lymphocyte activation
positive regulation of leukocyte activation
leukocyte proliferation
leukocyte migration
T cell activation
leukocyte cell−cell adhesion
mononuclear cell proliferation
lymphocyte proliferation
regulation of leukocyte proliferation
regulation of mononuclear cell proliferation
regulation of lymphocyte proliferation
regulation of inflammatory response
regulation of T cell activation
regulation of leukocyte cell−cell adhesion
positive regulation of cytokine production
leukocyte mediated immunity
positive regulation of lymphocyte activation
regulation of cell−cell adhesion
DNA−dependent DNA replication
negative regulation of cell activation
leukocyte chemotaxis
negative regulation of immune system process
negative regulation of leukocyte activation
leukocyte activation involved in immune response
chromosome segregation
myeloid leukocyte migration
adaptive immune response
cell activation involved in immune response
p.adjust
1.999444e−27
5.417003e−19
1.083401e−18
1.625101e−18
2.166801e−18
g
EnrichmentKEGG_UP
Cytokine−cytokine receptor interaction
Tuberculosis
Human T−cell leukemia virus 1 infection
Ribosome
Epstein−Barr virus infection
Phagosome
Cell cycle
Chemokine signaling pathway
Osteoclast differentiation
Lysosome
Hematopoietic cell lineage
Rheumatoid arthritis
Yersinia infection
Purine metabolism
Natural killer cell mediated cytotoxicity
TNF signaling pathway
Leukocyte transendothelial migration
Viral protein interaction with cytokine and cytokine receptor
Leishmaniasis
Fc gamma R−mediated phagocytosis
Chagas disease
B cell receptor signaling pathway
NF−kappa B signaling pathway
Toll−like receptor signaling pathway
p53 signaling pathway
Malaria
Pertussis
DNA replication
Primary immunodeficiency
Homologous recombination
GeneRatio
Count
p.adjust
h
EnrichmentGO_UP
antigen processing and presentation of peptide antigen via MHC class II
antigen processing and presentation of peptide or polysaccharide antigen via MHC class II
antigen processing and presentation of peptide antigen
antigen processing and presentation of exogenous peptide antigen
antigen processing and presentation of exogenous antigen
antigen processing and presentation of exogenous peptide antigen via MHC class II
antigen processing and presentation
humoral immune response
humoral immune response mediated by circulating immunoglobulin
regulation of lymphocyte proliferation
regulation of mononuclear cell proliferation
complement activation
immunoglobulin mediated immune response
regulation of leukocyte proliferation
B cell mediated immunity
protein activation cascade
negative regulation of lymphocyte activation
lymphocyte proliferation
mononuclear cell proliferation
adaptive immune response based on somatic recombination of immune receptors built from immunoglobulin superfamily domains
negative regulation of mononuclear cell proliferation
negative regulation of lymphocyte proliferation
negative regulation of leukocyte proliferation
negative regulation of leukocyte activation
leukocyte proliferation
negative regulation of cell activation
adaptive immune response
regulation of myeloid leukocyte differentiation
activation of immune response
positive regulation of antigen processing and presentation
p.adjust
i
EnrichmentKEGG_UP
Staphylococcus aureus infection
Antigen processing and presentation
Systemic lupus erythematosus
Tuberculosis
Complement and coagulation cascades
Th1 and Th2 cell differentiation
Hematopoietic cell lineage
Th17 cell differentiation
Cell adhesion molecules
Phagosome
Asthma
Intestinal immune network for IgA production
Inflammatory bowel disease
Graft−versus−host disease
Allograft rejection
Type I diabetes mellitus
Leishmaniasis
Autoimmune thyroid disease
Viral myocarditis
Rheumatoid arthritis
GeneRatio
Count
p.adjust

SFig.2 Differentially expressed genes analysis of rat DRGs at day3 after compression. a, **b**, c. Volcano plot showing the differentially expressed genes in L4 (**a**), L5 (**b**) and L6 (**c**). **d**, **f**, **h**. Paired enriched GO function terms in biological process of up-regulated genes in (**a**), (**b**), (**c**). **e**, **g**, **i**. Paired KEGG pathway enrichment of up-regulated genes in (**a**), (**b**), (**c**).

SFig.3

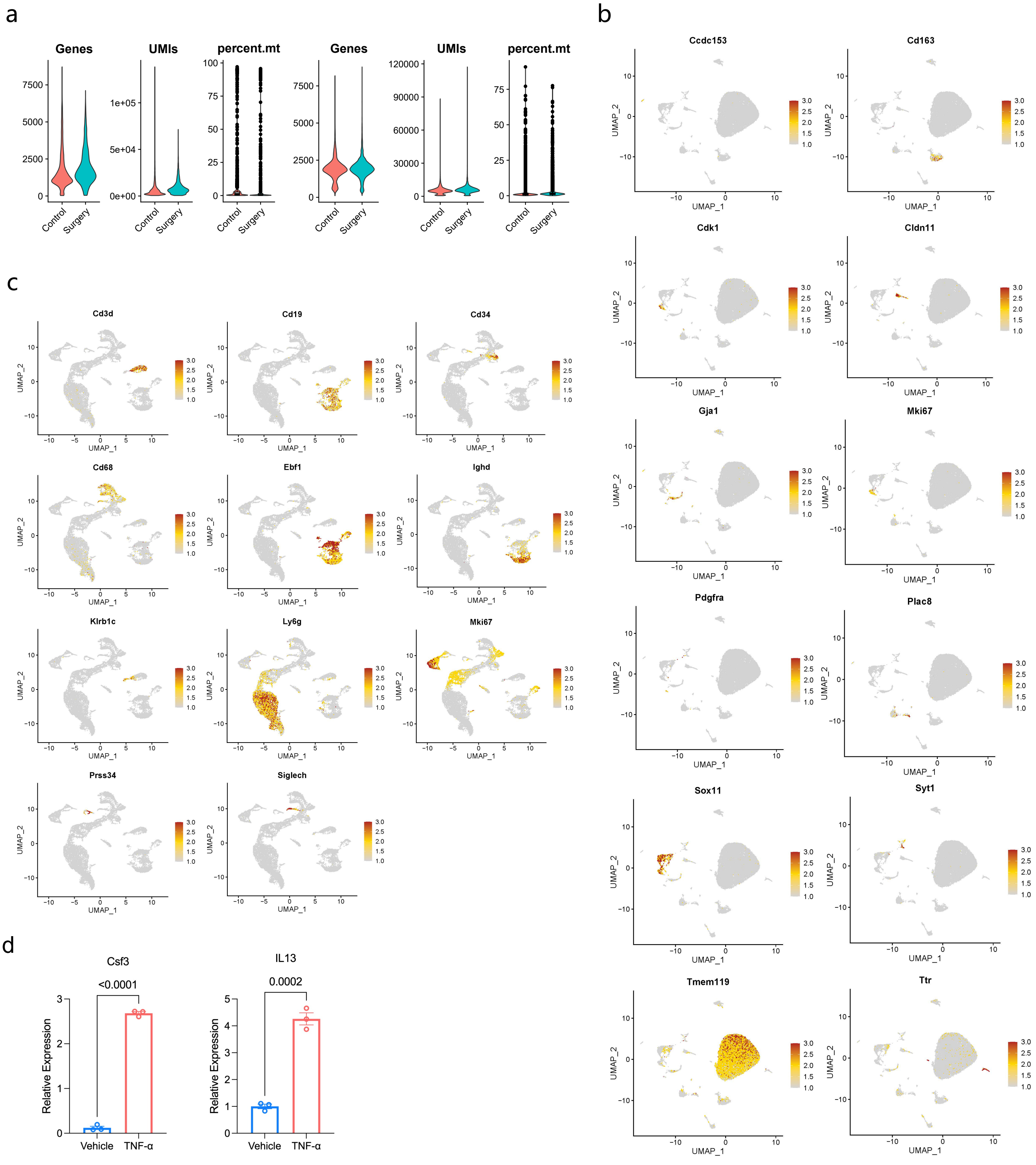

SFig.3 Quality control of single cell dataset. a. Number of genes, unique molecular identifiers (UMIs, transcripts) and percent of mitochondrial genes in bone marrow (left) and brain (right) datasets. **b**, **c**. Feature plot showing the expression of cell type specific marker genes in bone marrow (**c**) and brain (**b**) data. **d**. qPCR analysis of Csf3 and IL13 in bone marrow derived macrophage treated with or without TNF-α.

SFig.4

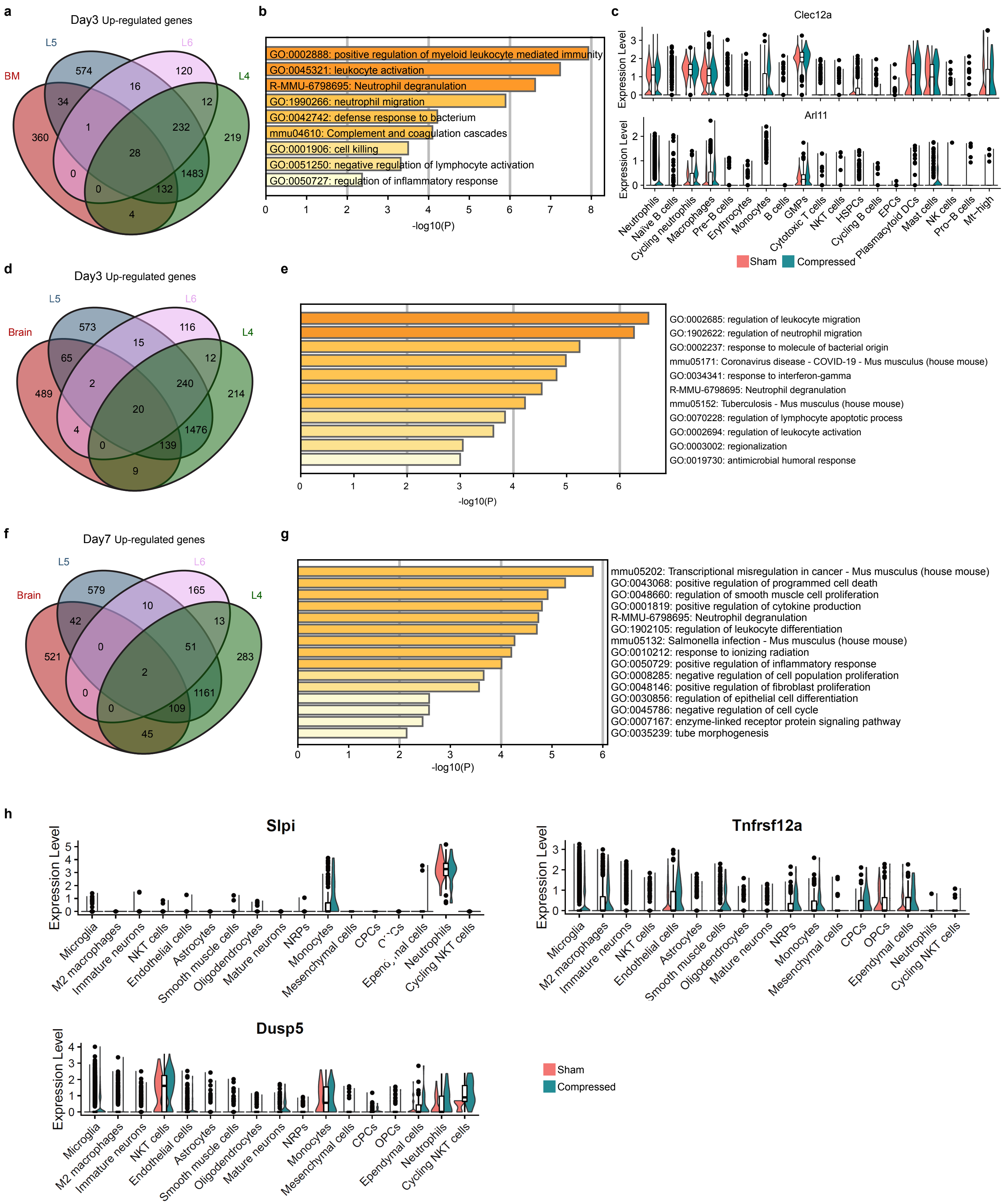

SFig.4 Comparative analysis of bone marrow and brain data between mouse and rats perturbed genes after compression. **a**.Venn plot showing the number of overlapped genes between rat L4-6 DRGs and bone marrow in day3. **b**. Paired enriched GO function terms in biological process of overlapped up-regulated genes in (**a**). **c**. Violin plot showing the expression of Clec12a and Arf11 in bone marrow cell types between sham and compression group in day3. **d**. Venn plot showing the number of overlapped genes between rat L4-6 DRGs and brain and mouse brain. **e**. Enriched GO function terms in biological process of mouse overlapped up-regulated genes with L6. **f**. Venn plot showing the number of overlapped genes between rat DRGs (L4, L5, L6) and mouse brain. **g**. Enriched GO function terms in biological process of mouse overlapped up-regulated genes with L6. **h**. Violin plot showing the expression of Slpi, Tnfrsf12a and Dusp5 in bone marrow cell types between control and compression group.

SFig.5

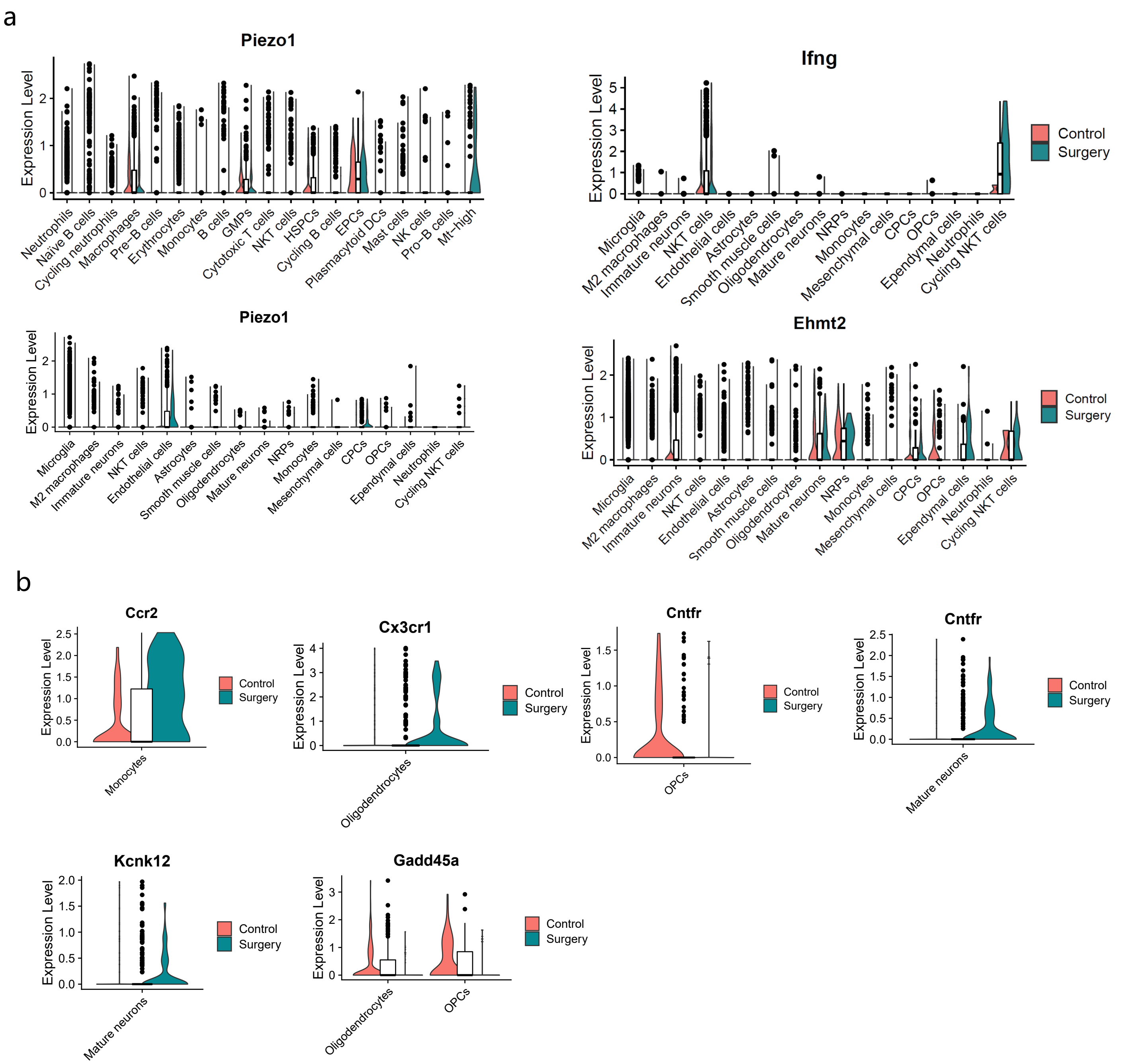

SFig.5 Expression of pain-regulated genes in brain cell type. a. Violin plot showing the expression of pain-threshold related genes in brain cell types between control and compression (surgery) group. **b**. Expression of pain-related genes in specific brain cell types.

SFig.6

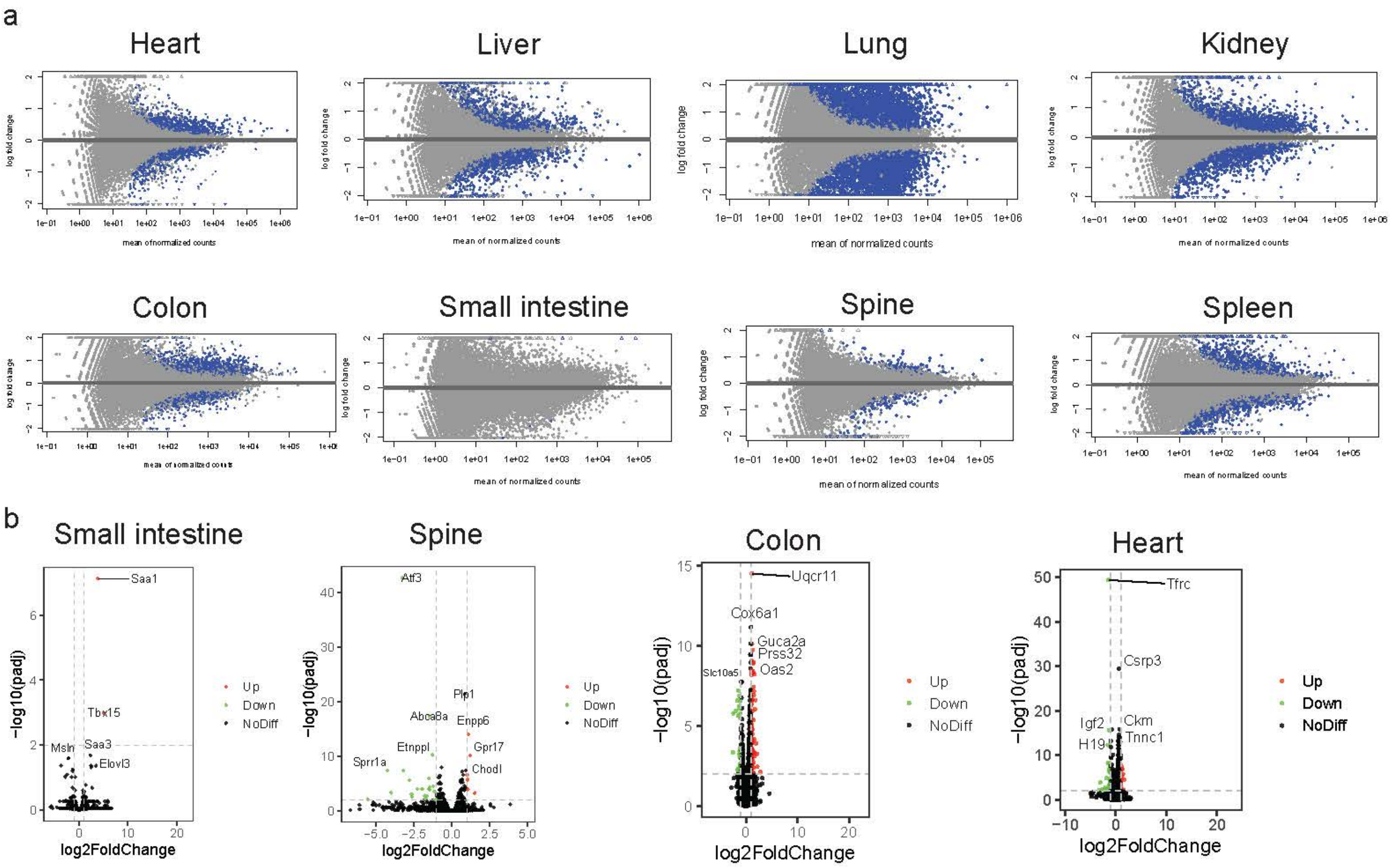

SFig.6 Cross-tissue transcriptome changes. a, M-versus-A plot showing the distribution of variable genes in different organs. **b**, Volcano plots showing the DEGs in tissues with modest changes.
